# Assessing the acceptability of incentivising HPV vaccination consent form return as a means of increasing uptake

**DOI:** 10.1186/s12889-018-5278-z

**Published:** 2018-03-20

**Authors:** Lauren Rockliffe, Amanda J. Chorley, Emily McBride, Jo Waller, Alice S. Forster

**Affiliations:** Research Department of Behavioural Science and Health, UCL, Gower Street, London, WC1E 6BT UK

**Keywords:** Vaccination, Reward, Adolescent, Papillomavirus vaccines, Motivation, Prevention

## Abstract

**Background:**

Uptake of human papillomavirus (HPV) vaccination is high overall but there are disparities in uptake, particularly by ethnicity. Incentivising vaccination consent form return is a promising approach to increase vaccination uptake. As part of a randomised feasibility trial we qualitatively assessed the acceptability of increasing uptake of HPV vaccination by incentivising consent form return.

**Methods:**

In the context of a two-arm, cluster randomised feasibility trial, qualitative free-text questionnaire responses were collected from adolescent girls (*n* = 181) and their parents (*n* = 61), assessing the acceptability of an incentive intervention to increase HPV vaccination consent form return. In the incentive intervention arm, girls who returned a signed consent form (regardless of whether consent was given or refused), had a 1-in-10 chance of winning a £50 shopping voucher. Telephone interviews were also conducted with members of staff from participating schools (*n* = 6), assessing the acceptability of the incentive. Data were analysed thematically.

**Results:**

Girls and parents provided a mix of positive, negative and ambivalent responses about the use of the incentive to encourage HPV vaccination consent form return. Both girls and parents held misconceptions about the nature of the incentive, wrongly believing that the incentive was dependent on vaccination receipt rather than consent form return. School staff members also expressed a mix of opinions on the acceptability of the incentive, including perceptions of effectiveness and ethics.

**Conclusions:**

The use of an incentive intervention to encourage the return of HPV vaccination consent forms was found to be moderately acceptable to those receiving and delivering the intervention, although a number of changes are required to improve this. In particular, improving communication about the nature of the incentive to reduce misconceptions is vital. These findings suggest that incentivising consent form return may be an acceptable means of improving HPV vaccination rates, should improvements be made.

**Trial registration:**

ISRCTN Registry; ISRCTN72136061, 26 September 2016, retrospectively registered.

## Background

A vaccination against human papillomavirus (HPV) was introduced in the UK in 2008. The vaccination is delivered primarily through schools and is recommended for girls aged 12 to 13 years old (in school Year 8). Since the introduction of the vaccine, HPV immunisation programmes have been implemented in 64 countries nationally, with an estimated 118 million females targeted [1].

In the UK, uptake of the vaccination is high, with 87% of girls receiving at least one dose of the vaccine in 2015/2016 [2], although there is considerable variation in uptake across the country, ranging from 68% in Brent, to 97% in Sunderland, and as high as 100% in areas with small populations [2]. Furthermore, ethnic inequalities in uptake have been consistently documented, which appear to be independent of deprivation [3, 4].

It is important that we devise approaches to improve uptake of the vaccination. In the UK and elsewhere in the world, there is evidence of assortivity of sexual mixing (sexual partnerships being more likely between people within the same ethnic group) [5, 6]. Whilst high uptake of the vaccine should offer herd protection to those who remain unvaccinated, such patterns of sexual mixing may exacerbate disease inequalities as unvaccinated individuals who engage in sexual partnerships with others who have not been vaccinated will not benefit from herd immunity.

One potential way of encouraging healthy behaviours, such as vaccination, is through the use of incentives, which have been successfully used to encourage behaviour such as attending non-smoking clinics, attending check-ups or completing screening [7, 8], although much of the research in this area has been conducted in the United States. Defined as a direct or indirect reward for attaining a goal [9], types of incentives may include the provision of gifts or prizes, lotteries, free or reduced price goods or services, or financial rewards such as cash, or cash-like rewards, such as shopping vouchers [10, 11]. Financial incentives have been found to be around 1.5 to 2.5 times more effective at encouraging healthy behaviours than no intervention or usual care [11]. In the context of vaccination, financial incentives have been used to encourage vaccination receipt, with varying success [12–15].

However, opinion on the use of financial incentive interventions varies widely; while some believe such incentives play an important role in the promotion of health behaviours, they can be viewed as a form of bribery or coercion, and are perceived by some to undermine individual autonomy [16, 17]. Financial incentives have been found to be less acceptable than other methods of behaviour change, such as education or peer support [18], although acceptability has been shown to increase with reported effectiveness [19]. If the financial incentive is considered to be an effective way to change behaviour and to be cost-effective, if it provides benefits to individuals and wider society, and is considered to be fair, then it is also more likely to be viewed as acceptable [20]. Furthermore, financial incentives in the form of food or shopping vouchers are viewed as more acceptable than cash incentives [17, 19], as it reduces the chance that the incentive is used to engage in negative health behaviours such as tobacco use or alcohol consumption [17].

These ethical concerns, combined with the fact that vaccination is not mandatory in the UK, suggests that incentivising vaccine receipt in itself is not an acceptable option for increasing HPV vaccination rates. An alternative option which has not previously been explored is incentivising the return of vaccination consent forms. This is an approach which has previously been recommended by adolescents, as a way to increase school-based vaccinations [21]. In practice, individuals under the age of 16 are usually required to have consent from a person with parental responsibility in order to receive a vaccination [22], although they may legally consent for themselves [23]. This is variably implemented. The HPV vaccination consent process involves girls delivering information about the vaccination and a consent form to their parent^1^. Parents are asked to give their child the completed consent form to return to the school, regardless of whether they are providing consent or refusing vaccination. The number of consent forms that are returned, for school-based vaccinations, has been shown to be improved if non-responsive parents are prompted with a second consent form [24]. Similar prompts have also been found to be effective at improving consent form return for HPV vaccination; it has been suggested that around 60% of HPV vaccination consent forms are returned to schools, unprompted [25]. Of the remaining 40%, half of these consent forms will be returned, consenting to vaccination, if followed up by a telephone call from an immunisation nurse [25]. This suggests that by increasing consent form return rates, vaccination uptake rates should show a concomitant increase.

Between July 2016 and January 2017, we conducted a randomised feasibility trial of an adolescent incentive intervention to increase HPV vaccination uptake by incentivising HPV vaccination consent form return. The key objectives of the trial were to assess the feasibility of a future randomised controlled trial (RCT) and to generate proof of concept evidence of the effect of the intervention on both consent form return and vaccination uptake, as well as any unintended consequences of the intervention, mechanisms of action and incentive acceptability [26, 27].

As with any intervention, successful implementation depends not only on the feasibility, but also on the acceptability. Acceptability is a necessary but not sufficient condition for intervention efficacy [11] and reflects “the extent to which people delivering or receiving a healthcare intervention consider it to be appropriate, based on anticipated or experienced cognitive and emotional responses to the intervention” [11]. We therefore collected qualitative data assessing the acceptability of the incentive, as part of a process evaluation embedded into the feasibility trial. In this paper, we present this qualitative data and aim to assess the acceptability of the incentive for adolescent girls, their parents, and participating school staff.

## Methods

The feasibility trial was conducted from July 2016 to January 2017 in London, UK. The trial was registered at ISRCTN (ISRCTN72136061) and has been fully described elsewhere [26, 27]. Ethical approval was granted by University College London Research Ethics Committee (6615/02).

### Feasibility trial

#### Trial context

The feasibility trial was carried out in schools based in the London boroughs of Enfield and Southwark, with an equal number of schools in each locale. In 2015/2016 uptake of the first dose of the HPV vaccine was 82.4% in Enfield and 89.7% in Southwark [2].

#### Participants

All secondary schools with female Year 8 students in Enfield, Southwark and Lambeth, were invited to participate in the trial (*n* = 60). Of those, 51 schools did not respond or declined the invitation. Nine schools were randomised (four incentive intervention arm, five standard invitation arm) but three schools withdrew before the trial started. In total, six secondary schools participated in the feasibility trial, three in each arm. A statistician used computer generated random numbers to allocate schools into each arm of the trial using blocked randomisation. Participants were Year 8 girls and their parents, and staff members from participating schools.

#### Intervention

The intervention has been described in full elsewhere [26, 27]. In brief, schools in the standard invitation arm of the trial provided Year 8 girls with standard information about the HPV vaccination and a consent form to be signed by their parent, and returned to school. Schools in the incentive intervention arm provided Year 8 girls with the same information and consent form, but offered them the opportunity to be entered into a prize draw to win one of several £50 Love2shop vouchers^2^ if they returned their consent form, signed by their parent. This was communicated to girls verbally by their form tutors and via a letter. Girls returning a signed consent form were entered into the prize draw regardless of whether the form said ‘yes’ or ‘no’ to vaccination. The prize draws were at the school level and eligible girls had a 1-in-10 chance of winning.

#### Trial procedures

Opt-out consent was gained from parents, for all girls and parents participating in the trial. Consent was gained after schools were randomised, but prior to vaccination day and delivery of the intervention. Selected staff members (including a school nurse, communications manager, pastoral officer, head of inclusion, attendance and welfare officer, and headteacher’s personal assistant) at the three schools in the incentive intervention arm of the trial were asked to inform Year 8 tutors about the incentive via email (asking them to pass the information on to girls in their tutor group) and additionally to Year 8 girls via a letter. The delivery of this information coincided with the distribution of HPV vaccination consent forms.

### Assessing acceptability of the incentive

#### Girls and parents

As part of the trial, girls and parents were asked to complete a questionnaire approximately one week after vaccination day. The questionnaires assessed unintended consequences of the intervention, possible mechanisms of action (these outcomes are reported in [27]) and attitudes towards the incentive. Attitudes were assessed using two free-text response acceptability questions and are the focus of this analysis. Girls from the incentive intervention arm of the trial answered the question *“What did you think about being entered into a prize draw to win a £50 voucher if you returned the HPV vaccine consent form?”* Parents from both the standard invitation and incentive intervention arms were provided with information about the aim of the trial and use of the incentive. They were then asked to respond to the question *“Please explain why you do or do not think it [the incentive] is a good idea.”*

#### School staff

Following the completion of data collection in each school, staff members who had been involved with the running of the trial were invited to participate in an interview. This invitation was also extended to other staff members, including Year 8 tutors and senior staff, although none chose to participate.

Interviews were conducted by one of two researchers (LR and EM) over the telephone with staff members from participating schools. EM conducted five of the interviews and had not previously been involved in the running of the trial. LR was involved in the running of the trial and conducted one interview with a staff member with whom she had had no prior contact. All participants provided verbal consent. A semi-structured topic guide was developed for use in the telephone interviews. The guide covered topics relating to the acceptability of the incentive, which is the focus of this analysis and the acceptability of the trial procedures, which is not discussed here. Topics assessing incentive acceptability included ‘attitudes towards the incentive’, ‘initial thoughts about taking part’, and ‘overall experience of participating in the trial’. Interviews lasted an average of 17.41 min (ranging from 10.52 min to 20.38 min), were audio-recorded and transcribed full verbatim.

### Data analysis

Data were analysed thematically by two researchers (LR and AC). The free-text questionnaire data and interview data were analysed separately.

#### Free-text questionnaire data

Initially, the girls’ questionnaire data were coded separately from the parents’ questionnaire data. Parents’ questionnaire data from the standard invitation and incentive intervention arms of the trial were coded together, owing to the small number of questionnaire responses received from parents in the standard invitation arm of the trial. Initial codes were generated by the two researchers (LR and AC). These were then refined by combining similar codes and a coding frame was developed and applied to the data by both researchers. Cohen’s k was calculated to determine the level of inter-rater reliability for the coding. There was moderate agreement between the coding of the girls’ questionnaire data (k = .711; *p* < .001) and weak agreement between the coding of the parents’ questionnaire data (k = .518; *p* < .001). Discrepancies were resolved through discussion and interpretations of the coded data were made by the research team. Given the similarity of emergent themes, both sets of coded data were interpreted together, although researchers remained aware of the source of each data fragment throughout this process.

#### Interview data

Initial codes were generated, line-by-line, by the two researchers (LR and AC). These were then refined by combining similar codes and re-applied to the data by both researchers, using the qualitative data analysis software Nvivo 11 [28]. Interpretations of the data were made by the research team and any discrepancies were resolved through discussion.

The results present the main themes derived from the data. The results from the analysis of interview data and free-text questionnaire data are reported separately. Quotes are used to illustrate the themes and are reported with participant codes that comprise school number (1–6), trial arm (A; incentive intervention arm, B; standard invitation arm) and participant identifiers (e.g. girl001/parent001/staff001). Themes unrelated to the aim of this paper have not been discussed.

## Results

### Sample characteristics

A total of 203/255 Year 8 girls completed a questionnaire in the incentive intervention arm (80%). Of those who responded, 181 free-text responses were provided to the acceptability question. The majority of responses were provided by girls who were Christian (50%) and born in the UK (91%). A total of 95/575 parents completed a questionnaire (17%; 35 standard invitation arm, 60 incentive intervention arm). Of these, 61 provided a free-text response to the acceptability question (19 standard invitation arm, 42 incentive intervention arm). The majority of these parents were mothers (64%), Christian (53%), were born in the UK (71%), and the most common ethnic group was non-White British (49%). Telephone interviews were conducted with six school staff members from four of the six participating schools (one standard invitation arm, three incentive intervention arm). The job roles of those participants included school nurse (*n* = 2), administrator (*n* = 1), communications manager (*n* = 1), pastoral officer (*n* = 1) and head of inclusion (*n* = 1). Table 1 details the number of participants who contributed qualitative data included in this analysis.

**Table 1:** Number of participants contributing qualitative data

	Year 8 girls (n)	Parents (n)	School staff members (n)
Incentive intervention arm			
School 1	56	6	1
School 2	68	23	3
School 3	57	13	1
Standard invitation arm			
School 4	–	4	1
School 5	–	15	–
School 6	–	–	–

### Free-text questionnaire responses from parents and girls

#### Misconceptions

Despite the offer of an incentive being contingent on consent form return, and not vaccine receipt, several girls and parents believed that the incentive was a way to encourage girls to have the vaccination and/or reported that it had motivated them/their daughters to have it. Regardless of this misunderstanding, these comments were mainly in favour of the incentive being used in this way.

*“I would have had the jab anyway but I know that a lot of people were more likely to have it because of the voucher” (2-A-girl074)*

*“It would help them to get the vaccination because of the prize they might win” (3-A-parent059)*

Ambiguous comments were also made by some girls which suggested that they had misunderstood the nature of the incentive; one girl commented that *“it really don’t matter who wins, it just means they did better”* indicating that they may have thought the incentive was offered as part of a competition. Another commented that *“lots wouldn’t be tricked into thinking they had a guaranteed win”*. Other girls reported thinking that the offer of an incentive was contingent on not crying or being brave during the vaccination process.

#### Positive responses to the incentive

Both girls and parents expressed positive views about the incentive and its use to encourage form return. Many of the girls reported feeling excited and happy about the chance to win a prize. Some girls described how they planned to spend the winnings, whether that was on themselves, or on family members.

*“I felt very excited and pleased to have this chance to win” (2-A-girl037)*

*“I thought it was a great opportunity to spend and treat myself” (3-A-girl006)*

Similarly, a number of parents felt that the incentive was a good idea and some parents mentioned the positive ways the winnings could be spent, including donating to charity, buying books or going shopping.

A number of girls felt that the incentive was an added bonus to the vaccination, something many of them were planning to do anyway. Other girls were positive about the type of incentive offered, including the value. Furthermore, some felt that the incentive made the vaccination a more positive experience and reduced the worry associated with it. One girl reported that *“it calmed my nerves”,* while another stated that *“if people are worried about the vaccine it may help them”*. This was mirrored by the views of some parents.

*“I think that the injections are a little bit scary and being entered into the prize draw makes it seem more fun and better” (2-A-girl012)*

*“It helps to take her mind off it” (3-A-parent098)*

Many parents and girls commented on the perceived effectiveness of the intervention. Some parents felt that the incentive would motivate girls to return their consent forms and a number of girls shared this view. Some girls stated explicitly that it had encouraged them to return their form quickly, whilst others commented more generally on the potential for the incentive to motivate them and/or their peers to return their forms.

*“It does encourage them to get the consent form signed” (2-A-parent006)*

*“I think it's a really cool idea and definitely encourages people to bring their forms back into school” (2-A-girl001)*

For some girls and parents, positive views on the use of the incentive were strongly related to the perceived value and importance of the vaccine itself, with some taking the attitude that anything that could possibly increase coverage was a positive thing. It is possible that such comments were related to the previously outlined misconceptions about the purpose of the incentive.

*“I think that it is a good idea because it will encourage girls to have the vaccination which will benefit them” (3-A-girl102)*

*“…Strongly in favour of vaccination programme. Do anything to get as many immunised as possible!” (2-A-parent034)*

#### Negative responses to the incentive

However, a positive view of the vaccination was not necessarily linked to a positive view of the use of an incentive to promote form return (or to promote consent to vaccinate, as some mistakenly believed). For these girls and their parents, the protective benefits of the vaccine meant that the incentive was needless or inappropriate. For some, the vaccination itself was viewed as incentive enough.

*“I think that it is unnecessary because the consent form is very important and the girls should know well enough that it's essential to bring it back to school” (2-A-girl072)*

*“People shouldn’t want to do the injection or bring in their forms for money, but for their own health” (5-B-parent096)*

Several negative responses were received from girls and parents alike. Some girls felt that the incentive was *“stupid”* or *“unnecessary”* and some had a preference for an alternative allocation of the prize. This view was mirrored by those of some parents, including one who felt that only girls receiving the vaccination should be eligible to receive the prize.

*“I think it's a good idea but slightly unfair as only some people win” (2-A-girl067)*

Others were concerned about the potential for the incentive to reduce informed choice regarding the vaccination, and several parents expressed a preference for more health education on the topic. There were responses from both girls and parents which described the incentive as a *“bribe”,* and encouraging vaccination for the wrong reasons. Views such as these appear to be due to misconceptions about the nature of the incentive, as previously discussed.

*“I think it's a good idea to encourage the girls, however seems like a "bribe". Perhaps more health education is required?” (2-A-parent007)*

Other reported reasons for disliking the incentive included negative opinions on the type of prize offered; some girls were unimpressed with the type of shopping voucher on offer and/or the £50 value. A couple of girls also lacked belief in the reality of the prize, commenting *“I thought it was fake”* and *“I didn’t think anyone would win”.* A number of parents were of the opinion that girls should have no say regarding the vaccination and that incentives should therefore not be directed at them.

*“As the 12 year old child still needs parental consent it is unclear why the form is not sent to/returned by the adult - no need to involve/bribe the child in this transaction surely?”(2-A-parent036)*

Additional negative feedback came from a few girls who reported negative emotions related to the offer of an incentive. One girl claimed to have felt more anxious after hearing about the prize draw, whilst another reported being upset when she did not win. Furthermore, some girls were discouraged by the odds of winning and several commented that they felt they would be unlikely to win the prize.

*“…It didn’t really motivate me because it’s so unlikely to win” (2-A-girl039)*

#### Ambivalence towards the incentive

There were also a number of responses which suggested ambivalence or indifference towards the incentive. A number of girls and parents stated that they would have returned the form or consented to vaccination regardless of the incentive, but still perceived the prize draw as an added bonus, while other responses suggested complete indifference, with a couple of girls claiming to have forgotten about the incentive entirely.

*“I would've brought the form in anyways without the £50 voucher prize draw. But I was interested in the voucher” (3-A-girl063)*

*“I forgot about it I was just concerned about the injection” (1-A-girl036)*

### Interview findings from school staff members

#### Perceptions of the incentive

Staff members reflected on their opinions of the incentive prior to carrying out the trial. Some had thought it was a *“really good idea”* and *“an added extra bonus”* that would definitely encourage the girls to return their forms. One staff member stated that the incentive would *“take away the negativity [of the injection], which is painful”*. However, others had believed that the use of an incentive was unnecessary as they already had good form return rates in their school.

*“...you were trying to, I think, improve the return of the forms by offering them a financial incentive, but from past experience I don't think you needed to do that because they’re pretty good at, erm, getting it done anyway”(2-A-staff003)*

Following the completion of the trial, there were mixed opinions regarding how effective they perceived the incentive to have been. Some believed that it had been effective in encouraging girls to return their forms, despite having no effect on parental decision making.

*“Definitely, definitely, definitely. I think that’s what, you know, made them bring… most of them bring, bring their, um, forms back, knowing that there was something to gain at the end of it, you know […] Actually, um, because… we can, we can never say what parents are going to do, because it is their decision about whether they are having it or not…” (1-A-staff006)*

However, those participants who felt from the start that the incentive was unnecessary, were still of this opinion.

Despite this it was largely believed that the girls had a positive reaction to the prize draw. As reflected in the comments from participating girls, some staff believed the girls were *“excited”* and *“buzzing”* about the prize draw, with some staff believing that it was the relatively large value of the voucher that increased the positive reception to the idea.

*“Um, they were, they were really keen actually… yeah, that, that was, um, quite a big… because it was quite a big prize actually, so I think, yeah, they were, they were so pleased” (3-A-staff004)*

However, the reactions of the students were not unequivocally positive in all schools, with one participant reporting that the girls did not talk about the prize draw and that it made no difference to form return rates. Another participant reported issues with girls believing they were being bribed into having the vaccination, as has been previously discussed.

Furthermore, there was also some concern from one member of staff about the ethics of using a financial incentive to encourage form return, especially given the relatively high value of the voucher.

*“I don't know. I'm not entirely sure, umm, ethically, whether it's the right thing or not, but, y'know. It was quite interesting […] But, I'm not sure, really, in the scheme of things, whether it's the right thing, to persuade children to, y'know...” (2-A-staff001)*

## Discussion

The purpose of this paper was to assess the acceptability of an incentive intervention to encourage the return of HPV vaccination consent forms for adolescent girls, their parents and participating school staff members. The aim of the incentive was to indirectly increase HPV vaccination uptake by improving the number of consent forms returned in schools. Our analysis of free-text questionnaire data identified a mix of views from girls and parents regarding the acceptability of the incentive; Positive, negative and ambivalent responses were expressed by girls and their parents, and both these groups of participants held misconceptions about the nature of the incentive. The analysis of interview data with school staff members also highlighted a mix of opinions on the acceptability of the incentive, which included perceptions of effectiveness and ethics.

Sekhon, Cartwright and Francis [29] have proposed a theoretical framework that can be used to guide the assessment of acceptability of interventions from both a recipient and deliverer’s perspective. The framework comprises seven component constructs to assess acceptability and we have interpreted our results using these constructs as a guide; Affective attitude (how an individual feels about the intervention) was relatively positive for most school staff members, who believed the intervention was a good idea and felt that girls had responded positively. In terms of the ethicality of the intervention (the extent to which the intervention has a good fit with the individual’s value system) one school staff member questioned the appropriateness of using an incentive and a number of parents and girls reported feeling as though the incentive was being used as a bribe. However, this view was often linked to misconceptions about the nature of the incentive. Related to this, intervention coherence (the extent to which the individual understands the intervention and how it works) was not always high, as some girls and parents believed that the incentive was dependent on vaccine receipt, not consent form return. With regard to perceived effectiveness, school staff members were divided. However, those that felt it had been ineffective did so because of the high form return rates they ordinarily have at their school, not due to the design of the intervention. Many girls and parents felt that an incentive was an effective way to encourage form return. The constructs, opportunity costs (the extent to which benefits, profits or values must be given up to participate), burden, and self-efficacy, were not discussed by participants in relation to the incentive itself, suggesting they play less of a role in the perceived acceptability of the incentive in this context. Based on this framework the incentive intervention was found to be moderately acceptable to both the recipients (girls and parents) and deliverers (school staff members^3^) of the intervention, although it is evident that some improvements could be made to improve overall acceptability and clarity of its purpose.

The findings support previous research which has found that financial incentives may be viewed as a bribe or form of coercion [16, 17], a concern that a number of participants expressed. However, this concern was often based on the misconception that the incentive was dependent on vaccination receipt, rather than consent form return. This is an important finding, as the concept of the intervention is called into question if participants misunderstand how the incentive works. It is therefore vital that communication about the nature of the incentive is clarified and/or simplified. This may consequently improve acceptability of the incentive. This might be achieved by amending the wording of the letters given to girls via the schools or by clarifying the verbal instructions tutors are requested to deliver to girls in their tutor groups. Future qualitative research with girls may be beneficial to help us understand specifically which part of the information is misleading or easily misinterpreted, and to identify how we can better communicate the details of the incentive.

As previously discussed, incentivising vaccination receipt has ethical implications that mean it is not likely to be an acceptable option for improving HPV vaccination rates [16–18]. Our findings demonstrate that incentivising consent form return instead of vaccine receipt, is a moderately acceptable form of intervention for those receiving and delivering the incentive, which may have implications for the types of interventions used, not only within the HPV vaccination context, but potentially within the context of other school-based vaccinations. However, a number of improvements would be required in order to increase the acceptability of the incentive.

Furthermore, our findings suggest that for some girls, the incentive could lessen feelings of worry and fear about the vaccination, and make the process more positive. Experiencing fear and anxiety about the vaccination is common for adolescent girls within a school context and in some instances this can result in vaccination refusal [30]. The use of the incentive may therefore have the potential to improve vaccination uptake, not only by increasing the number of forms that are returned consenting to vaccination, but by lessening the fear experienced by some girls who might otherwise refuse the vaccination at the point of receipt. Bernard et al. [30] also found that parents were often aware of their daughter’s fear about the vaccination, which may potentially act as a barrier to providing consent. Lessening fear amongst girls, via the use of the incentive, may also help to mitigate this situation and help to facilitate consent provision from the point of view of the parent.

However, it is important to acknowledge the ethical implications of delivering an intervention such as this. Although the potential for serious harm appears to be relatively low, there are a number of issues that need to be considered. There may be the potential for girls to become distressed if they do not win the prize, and the results show that for some girls this was the case. Furthermore, girls may become distressed if their parent is unwilling to sign the form. Relatedly, it is important to consider if such disappointment may manifest into negative attitudes or behaviour towards prize winners. Other concerns include the possibility that some girls may forge their parent’s signature in order to be entered into the draw, or that parents may have grievances about the incentive being offered to girls, as opposed to parents, or about what the voucher is spent on. In order to identify whether such concerns are warranted and to assess the impact of such issues, further work with both girls and parents will be required.

Based on the coherence of participants’ responses with previously theorised aspects of intervention acceptability [29], we feel confident that making improvements to the way in which the nature of the incentive is communicated would likely increase acceptability of the incentive. However, there is reason to remain cautious; five out of the six interviews conducted with school staff members were with those who worked at schools randomised to the incentive intervention arm of the trial. Schools in this arm may have had more positive attitudes towards the incentive, than those working in schools randomised to the standard invitation arm, due to observing the impact of the incentive in their schools. A further limitation is that only 17% of parents responded to the questionnaire. The views of these participants are therefore not representative of all parents who took part in the trial and there may be a response bias towards those who felt more strongly, or more negatively, about the intervention. Due to the low response rate, data collected from parents in both arms of the trial were interpreted together. We were therefore unable to compare differences in parental attitudes between trial arms, which is an additional limitation. Furthermore, there was weak inter-rater reliability for the coding of the parents data. However, all discrepancies were resolved through discussion before the coded data were interpreted. The acceptability of incentive interventions has been found to be dependent on the context in which the intervention is delivered [31]. It is therefore important to be mindful that the incentive was trialled in only one context, in UK schools, and that the acceptability of the incentive may therefore differ if implemented in other settings, such as non-UK countries or outside of the school environment.

## Conclusions

Our findings indicate that the use of an adolescent incentive intervention to encourage the return of HPV vaccination consent forms is moderately acceptable to adolescent girls, parents and participating school staff members. However, changes would be required to improve this. In particular, communication about the nature of the incentive would need to be improved to reduce misconceptions about the purpose of the incentive. These findings will be used to guide the design of a future RCT, to increase acceptability of the incentive for those receiving and delivering the intervention. Incentivising consent form return could be an acceptable means of increasing uptake of HPV vaccination and potentially other school-based vaccinations, should improvements be made.

## Abbreviations

HPV: Human papillomavirus
NHS: National Health Service
RCT: Randomised Controlled Trial

## Footnotes

1 Where the term ‘parent’ is used, we are referring to any adult with parental responsibility.

2 Love2Shop vouchers are gift vouchers that can be spent in a range of high street shops, restaurants and major attractions.

3 By ‘deliverers’ we refer to all school staff members who participated in the trial, in both the incentive intervention arm and standard invitation arm. There was only one staff member in the standard invitation arm who participated in an interview. Despite not delivering the intervention, they provided feedback on the use of such an incentive.
